# A Scoping Review of Pressure Measurements in Prosthetic Sockets of Transfemoral Amputees during Ambulation: Key Considerations for Sensor Design

**DOI:** 10.3390/s21155016

**Published:** 2021-07-23

**Authors:** Siu-Teing Ko, Fredrik Asplund, Begum Zeybek

**Affiliations:** 1 Research and Innovation, Össur, 110 Reykjavík, Iceland; 2 Department of Machine Design, KTH Royal Institute of Technology, 10044 Stockholm, Sweden; fasplund@kth.se; 3 Healthcare Innovation Centre, School of Health and Life Sciences, Teesside University, Middlesbrough TS1 3BX, UK; b.zeybek@tees.ac.uk

**Keywords:** transfemoral socket, pressure sensor system, prosthetic socket comfort, gait monitoring, lower limb prosthetics

## Abstract

Sensor systems to measure pressure at the stump–socket interface of transfemoral amputees are receiving increasing attention as they allow monitoring to evaluate patient comfort and socket fit. However, transfemoral amputees have many unique characteristics, and it is unclear whether existing research on sensor systems take these sufficiently into account or if it is conducted in ways likely to lead to substantial breakthroughs. This investigation addresses these concerns through a scoping review to profile research regarding sensors in transfemoral sockets with the aim of advancing and improving prosthetic socket design, comfort and fit for transfemoral amputees. Publications found from searching four scientific databases were screened, and 17 papers were found relating to the aim of this review. After quality assessment, 12 articles were finally selected for analysis. Three main contributions are provided: a de facto methodology for experimental studies on the implications of intra-socket pressure sensor use for transfemoral amputees; the suggestion that associated sensor design breakthroughs would be more likely if pressure sensors were developed in close combination with other types of sensors and in closer cooperation with those in possession of an in-depth domain knowledge in prosthetics; and that this research would be facilitated by increased interdisciplinary cooperation and open research data generation.

## 1. Introduction

Even if emphasis is placed on preserving limb length [1], transfemoral amputations make up a substantial part of all amputations. Unfortunately, rehabilitation is especially difficult for transfemoral amputees, and research indicates that a high amputation level is associated with both prosthetic non-use and a decreased probability of remaining ambulatory [2,3]. Socket fit has been cited as one of the main factors affecting gait re-education, rehabilitation, and quality of life for amputees from the viewpoint of both amputees and clinicians [4,5,6,7,8,9]. Poor socket fit is the cause of at least one gait deviation, in people with lower limb amputation, which could be linked to premature long-term musculoskeletal degenerations [6,10,11]. The importance of a well-fitting socket is highlighted by the fact that several patient evaluation questionnaires, which aim to measure the success of prosthetic devices, specifically refer to socket fit and comfort [12,13].

To solve this problem, sockets—which couple the residual limb and prosthesis—must provide sufficient support and stability during activities of daily living. This is complex as it largely depends on the inter-related performance of socket design, liner properties, suspension mechanisms, and prosthetic alignment [14]. Large research efforts have thus been dedicated to prosthetic socket design [15], and there are many different design philosophies for prosthetic sockets [16]. There are two main socket types: total surface bearing (TSB), which aims to distribute the load evenly across the residual limb; and specific surface bearing (SSB) regions, which attempt to apply load through tolerant areas and hence off-load more sensitive regions [16,17,18,19]. For transfemoral amputees, these are most commonly ischial containment (IC) or sub-ischial–ramal containment sockets and quadrilateral (QUAD) sockets, respectively. IC sockets are considered to provide gait stability during single-limb support, which is partially achieved through TSB and by encasing the ischial tuberosity and ramus within the socket. In QUAD sockets, the ischium is outside of the socket, and weight is bored through the ischium on the socket brim, often resulting in gait instability [15,18]. Localized pressures from the socket are often causes of rejection by the patient; for example, on the medial ramus of IC sockets and on the perineum of QUAD sockets [18]. For comparison, in transtibial amputees, the patellar-tendon bearing (PTB) is a commonly prescribed type of TSB socket. This relies on the localized loading of the patellar ligament to off-load more sensitive regions, such as the fibula head and tibial crest [16,17,19,20].

The inability of a socket to perform as expected can be highlighted through changes in pressure distributions at the interface between the residual limb and socket—caused by normal daily residual limb volume fluctuations, soft tissue adaptation during post-operative recovery, and edema, for example [14,21]. The effect of such residual limb volume changes on socket fit is known to result in pain, skin disease, and gait instability [10,11]. Whereby it has been reported that transfemoral residual limbs may exhibit larger volume fluctuations compared to transtibial residual limbs [21]. Traditionally, and still predominantly, prosthetists utilize their experience and expertise to optimize socket design based on each individual patient’s characteristics, such as residual limb features and activity level [15]. The current state-of-the-art employs the use of additional sock plies to accommodate for both daily volume changes and post-operative residual limb changes [14]. Socks are worn over the residual limb when the socket is too loose or removed when the socket is too tight. However, this only uniformly alters the volume and is inconvenient and not always adhered to by prosthetics users [11,14,22,23,24].

To address this issue, adjustable sockets have been developed in the field with both smart and manually controlled systems [9,14,22,25,26,27]. Pressure—whether qualitative or quantitative—is often the prescribed scale against which adjustable socket volumes are altered. For example, one of the aims of adjustable sockets is to reduce pressures and shear stresses at the stump–socket interface. In line with traditional socket design theory, the adjustable socket adapts to equally distribute pressure across the residual limb, or relieves pressure from sensitive regions by exerting more pressure through more tolerant regions [14,23,27,28,29]. Furthermore, continuous socket volume adjustments have been shown to promote residual limb volume management [9]. More generally, continuous monitoring, such as pressure and temperature at the stump–socket interface, can provide immediate feedback on socket fit [4], which would enable more informed and timely intervention prescriptions by clinicians [30,31]. Thus, there has been growing interest in the development of such monitoring and sensing technologies to complement current clinical practice approaches to help overcome challenges in prosthetic use [31,32]. Indeed, pressure measurement and the mapping of lower-limb prosthetic sockets has already improved the understanding of prosthetic fit at a very fundamental level and has helped to facilitate objectively based socket designs [15,33]. During the last 50 years, a variety of measurement techniques have been employed in an effort to identify sites of excessive stresses [32,34,35]. The objectives of this experimental research into the measurement of socket stresses were to improve the level of understanding of the stump–socket interface [36], to evaluate the influence of prosthetic design parameters and alignment variations on the interface stress distribution, and to evaluate the quality of prosthetic fit [37,38]. Transfemoral amputees specifically, may thus benefit from the introduction of sensors to measure stress changes in both normal and tangential directions at the residuum–socket interface, both of which may be damaging to the soft tissues of the residuum [39].

Arguably, the evolution of pressure sensor systems for this purpose has been limited by the technical difficulties in addressing the demanding requirements of the *day-to-day* monitoring of the comfort and fit of prosthetic sockets. Walking in a laboratory and walking in “real life” is often not the same [40], and clinical tools will always be a niche market in comparison to technology used in consumer goods. However, these technical challenges are being overcome due to the emergence of wearable, cheap, and technically sophisticated sensing technology for health monitoring [41,42,43]. Breakthroughs, tested in the smart health domain, have occurred in wireless technology for data-intensive applications [44], power harvesting [45], security [46], analytics [47], rapid prototyping and manufacturing [48], and materials [49]. The likelihood that easily deployed, cheap, and reliable pressure sensors systems for clinical use will emerge has thus drastically increased during the last few years, even if several challenges remain.

One of the main challenges in the development of new and improved prosthetic technologies is the lack of practical inner socket sensors for monitoring the environment between the skin and the liner or socket [37], or the region between the residual limb and the socket [50]. Selecting suitable transducers is also challenging and relies on the specific experimental and clinical environment [30,37,51]. In general, four main types of sensors are used for pressure measurements in the socket: strain gauges and piezoresistive, capacitive, and optical sensors [32]. A review of research on interfacial stress measurements at the stump–socket interface of transtibial amputees found many merits in sensor development over the past 50 years; for instance, high sensitivity, reproducibility, higher spatial resolution, lightweight, ease of use, availability in various shapes and sizes, and the capability of conforming to irregular shapes. However, while some of these sensors provide valuable information, they are disadvantageous due to their bulkiness, weight, inflexibility, cost, and laborious integration methods with the socket [38,50]. In lower limb prostheses, measurements and mapping pressures to amputees’ anatomy are also commonly performed using commercially available, semi-flexible pressure monitoring systems such as the F-Socket (Tekscan, Inc., South Boston, MA, USA) [34,36,52,53,54] or Pliance^®^ system (Novel^®^ GMBH, Munich, Germany) [55,56]. While these systems do not require socket modification, they are still spatially limited [4], cannot be readily integrated into existing prosthetic components [50], and are not sensitive to shear stresses [39]. To be able to overcome some of the shortcomings associated with currently available pressure monitoring systems, textile-based sensors have also been studied due to their compliance and breathability, and relatively simple fabrication methods [50]. Moreover, to be able to eliminate the dependency on bulky, rigid components inside the socket, and also to be able to support continuous, long-range wireless communication to standard consumer electronics, conformable sensors have also been investigated [4].

Furthermore, this particular application of pressure sensors is a highly specialized endeavor, and transfemoral amputees are a specific cohort with many unique characteristics compared with those with other levels of amputation. Many of the previously mentioned research developments for lower limb prosthetics were solely tested with transtibial amputees [4,10,11,23,50]. Several review articles on lower limb socket technology do not explicitly discriminate their findings against transfemoral or transtibial prostheses, but all instances cited more articles whose titles referred to the terms transtibial or below-knee compared with the terms transfemoral or above-knee, with a roughly 2.4 times higher prevalence on average [9,15,32,36,57]. This is further emphasized by another review article that analyzed interface stress measurements in transtibial sockets only [38], but similar analyses have not been published for transfemoral amputees; thus, the data for transfemoral amputees are very limited [15]. It is thus unclear whether existing findings regarding the use of pressure sensors to improve comfort and fit are applicable, or whether associated studies are conducted in ways that are likely to lead to substantial breakthroughs. This is an especially pressing concern for studies with an experimental design, as these should reconcile multiple research and industry disciplines to arrive at valid results. This is further aggravated by the lack of research that is clinically powerful—for transfemoral prosthetic socket comfort and fitness—due to the low methodological quality of the study design in numerous biomechanical and patient-centric studies [9].

To investigate whether the use of pressure sensors in prostheses is relevant for transfemoral amputees and whether there is any possibility to help guide methodological choices in future studies, we surveyed the existing literature in a scoping review. The review was reported following the relevant extension to the PRISMA guidelines [58,59]. The focus was to map the existing research on the use of pressure sensors to support transfemoral amputees and identify knowledge gaps as well as the strengths and weaknesses in the associated study designs. In this study, we therefore aimed to address the following research questions:

1. Is there a de facto methodology for experimental studies of the implications of intra-socket pressure sensors used by transfemoral amputees?
2. How should sensors be designed for application in prosthetic sockets to monitor transfemoral amputees for evaluating comfort and fit?

Answering these questions should facilitate the emergence of sensing, monitoring, and actuator technologies, specifically for the application of lower limb prosthetics, with the potential to significantly improve socket design and provide effective residual limb health monitoring.

## 2. Methodology

We conducted a comprehensive scoping literature review of all information related to pressure measurements in the prosthetic sockets of transfemoral amputees, following the reporting guideline by Tricco et al. [59]. Further guidance from earlier work [60] on conducting scoping reviews was also adhered to throughout the assessment, ensuring that the investigation was thorough and transparent. We note that, as highlighted by previous literature reviews [61,62], scoping reviews differ from systematic reviews in that they aim to answer broader types of research questions [58].

### 2.1. Information Sources, Search Strategy and Eligibility Criteria

The four databases Science Direct, Web of Science, PubMed, and Scopus were searched. This choice of information sources was made to cover a wide, high-quality range of journals and disciplines, while at the same time making sure to cover relevant subject areas such as biomedicine and engineering. The combinations of keywords used for the literature search are summarized in Table 1. The search was first completed in March 2019 and then repeated in June 2020 to include newly published articles.

**Figure 1:**
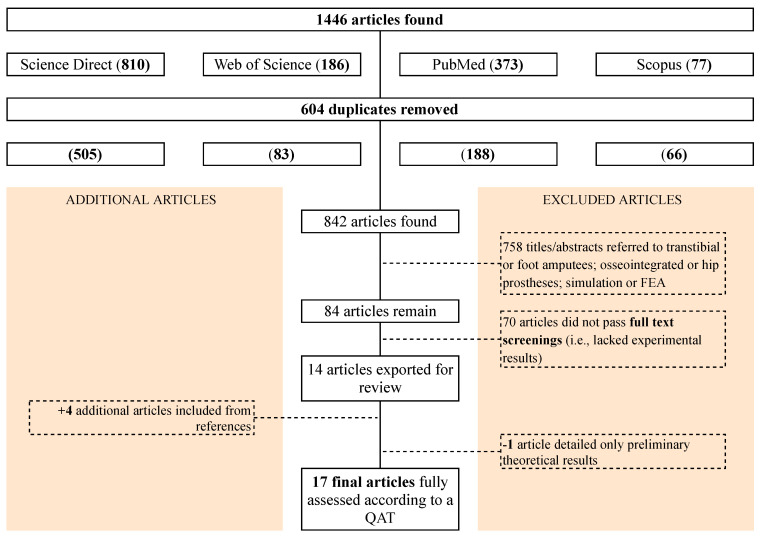
Flow diagram outlining study.

Publication dates and journals were not used to exclude research papers, as the research questions were broad. Search criteria #2 and #6 yielded a vast number of results from Science Direct and Web of Science due to the ambiguity of the keywords. The associated results were therefore excluded entirely. Due to the other combinations of keywords, the authors believe this has only added a small risk that relevant publications have been unintentionally excluded. Grey literature was also excluded. The overall structure and most important details of the protocol carried out during the study are visualized in Figure 1.

### 2.2. Selection and Critical Appraisal of Sources of Evidence

The identified literature from all four databases was collated. After duplicates were removed, 842 papers were identified for consideration. Two further screening processes were conducted. Firstly, the titles and abstracts that referred to transtibial or foot amputees, osseointegrated or hip prostheses, simulations, or finite-element analysis (FEA) were excluded. This was done to remove publications that did not address transfemoral amputees, who are the main cohort focus of this review. To avoid misunderstandings during the analysis, papers in languages other than English were also excluded. At this point, 84 articles remained. At the next selection level, 70 articles were eliminated based on a full-text screening to identify and discard papers that did not present experimental results. To identify whether a de facto methodology existed, only experimental procedures were of interest in this review. Within these 70 articles, some articles that should have been eliminated during the first screening process (i.e., that did not relate directly to transfemoral amputees) were missed and were therefore removed during the full-text screening. Papers that were unavailable as full texts were also eliminated. To further ensure that no relevant sources were missed, relevant publications that were referenced in the remaining papers were appended at this stage. This resulted in 17 papers eligible for further consideration.

The remaining papers were subjected to a critical appraisal based on a suitable quality checklist defined in a Cochrane Review guideline [63]. All three reviewers took part in the appraisal. This quality checklist was originally developed to evaluate randomized controlled trials. As the identified papers were mostly single-subject designs, the quality checklist was tailored for improved relevance. This modification was based on a discussion of each quality criterion by the three reviewers prior to the assessment. This ensured that the quality appraisal was calibrated and any differences in interpretation between the reviewers were minimized. The modified quality checklist is presented as a Quality Assessment Table (QAT), described in Table 2. It comprises 10 criteria, which are categorized into four groups: hypothesis, patient selection, intervention and assessment, and statistical validity. For each paper, each criterion was scored “1” if it was sufficiently fulfilled, “0” if it was insufficiently fulfilled, or “N/A” if the criterion was deemed irrelevant. The table also included an option to document the part of the paper in which the criterion was fulfilled and a section where comments or queries could be noted.

Each reviewer independently assessed each paper so that they would not be influenced by the evaluations of the other reviewers. The completed QATs were then compiled and the results were compared. Contradicting results between the three reviewers were highlighted and discussed in depth, and scores were adjusted to reach an agreement between the reviewers.

The grading system presented in Table 3 was introduced to control for intervention and measurement bias. It was designed to highlight the papers that answered the research questions of this scoping review, as well as to eliminate articles that lacked sufficient quality. For example, in order to achieve an A or B grade, a paper needed to fulfill point X because it is well-established that a good quality research paper should clearly identify the aim of the study. To help answer the research questions of this review, it was important to be able to draw cross-study comparisons; thus, a sufficient description of the experimental intervention was required. This was enforced by the fulfillment requirement of all points in group A as well as B5 and B9 to obtain an A grade, while only A1 and B9 were required for a B grade. The outcome of the grading system allowed papers graded A to be considered without any concerns, while papers graded C and D were excluded due to the low quality in regard to the purpose of this review. Papers graded B were included for further consideration, but the analysis of these papers included only the criteria that were evaluated to be sufficient. Overall, this process ensured that weakly supported statements were not included in the analysis.

### 2.3. Data Extraction and Synthesis Methodology

As a result, 12 research papers were graded A or B. These are shown in Table 4 and were subject to content analysis. A summary of the articles that did not pass the quality assessment is presented in Table A1 in Appendix A.

As the differences between the papers were complex, the analysis and synthesis to answer the research questions required a fine-grained content analysis rather than a summary of high-level characteristics of the studies. Therefore, data extraction took place in three steps. Firstly, each paper was coded by one of the reviewers; i.e., text sections, mostly separate sentences and paragraphs, were analyzed and labeled with a word or short phrase (*a code*). To avoid reviewer bias, this was not the reviewer who carried out the initial search and screening process. Coding was first descriptive [75]. In descriptive coding, each code aims to capture the topic of the text section; i.e., what is talked or written about. The purpose of the coding was thus to inductively capture specific attributes or characteristics of the study; for example, *“test subject profile given”* and *“sensors calibrated”* are two separate codes used in this study [75]. Each code was entered into a codebook together with a short explanation and a note regarding in which papers it appeared. Secondly, the codebook was reviewed and codes appended by the other two reviewers to ensure completeness. This resulted in approximately 100 descriptive codes. In this way, each new instance of a code could be checked against previous instances in other papers as well as establishing its meaning in the codebook. This ensured that the coding was consistent and stayed close to the intention of the surveyed papers. Thirdly, the coding then focused on identifying patterns with regards to the aim of the study [76]. In this investigation, patterns were based on groups of codes with a related meaning. For example, codes illustrating the technical characteristics of the sensors and codes describing patient characteristics are two separate patterns. Patterns were then combined into separate *themes*.

Finally, these themes were discussed by the reviewers. This discussion served to ensure that the identified themes remained true to the content of the surveyed papers and were not the subjective interpretation of a single researcher.

## 3. Results and Analysis

Several themes were established upon completion of the literature search, critical appraisal, and data extraction and synthesis process. Five of these themes were found to have a bearing on the research questions this paper sought to address. They are as follows: the importance of biomechanical hypotheses, diversity of domain-dependent knowledge, assessment of sensor type and use, sensor placement, and data handling and presentation. To ease the discussion, the themes are separated into and presented as belonging to two different categories in this section—research landscape and study attributes.

### 3.1. Research Landscape

Themes in this section refer to the foundations for the scientific research and relate to the decision process behind study design.

#### 3.1.1. Importance of Biomechanical Hypotheses

Most papers had an inductive approach, eschewing hypotheses in favor of a priori data analysis. Some studies had an explicitly stated purpose that was more aligned with pure engineering than scientific inquiry [69]. Even if this led to interesting results in the papers graded A or B, the strengths of a deductive approach in this context were clearly demonstrated by a minority of the papers.

Only one code related to this pattern (Table 5).

#### 3.1.2. Diversity of Domain-Dependent Knowledge

Although the papers were written by experts from vastly different domains, such as engineering and health sciences, there were few substantial references to domain knowledge. This is problematic, as it is difficult to know whether the studies addressed the most important topics with a realistic approach. Some important observations were only mentioned by a single study: that the stress can be considerable when donning a prosthesis [67], that comfort is a complicated construct and not simply associated only with high pressure [73], and that there are various different mechanical challenges (i.e., bending, torsion, elongation) to specific types of polymer-based sensors placed in prostheses [69]. Furthermore, the hypothesis that shear might be as important, or more important, than orthogonal pressure was noted by only three studies.

Similarly, a majority of papers gave comprehensive descriptions and detailed summaries on patient specific characteristics (e.g., age, weight, general stump condition, and activity level, etc.). Despite the mentioning of these qualities in the methodology sections, only a few papers discussed or addressed their impact on the heterogeneity of the outcome results.

In many papers, subject-specific profiles were further detailed with definitions of, for instance, prosthetic type, socket type, and self-selected walking speed. Some studies used duplicates of the patients’ own sockets, allowing test subjects to accustom themselves to the trial sockets before measurements began, mentioning this as a warm-up procedure. Only a few considered the importance of patients getting used to the socket (duplicate or their own) for a sufficient period before the data collection started. Only three research groups designed their studies with planned rests to avoid fatigue during data collection.

The codes relating to this pattern are summarized in Table 6.

### 3.2. Study Attributes

Themes presented here relate to specific components of the studies undertaken in the publications. For example, they include technical descriptions of the sensors used in the studies and the methods of presenting the data acquired.

#### 3.2.1. Assessment of Sensor Type and Use

Approximately half of the reviewed papers assessed whether the sensor type employed was suited to the purpose of the study; for instance, with regards to sensitivity, non-linearity [39,64,68], hysteresis [39,64,68], creep [64,65,69], error range [39,64,65,66,67,68], and measurable range [39,66,68]. There were large differences in what was perceived as suitable, but examples included for instance <5% for non-linearity errors [39], <1% of the full-scale operating range for hysteresis errors [39,68], and <1% for instrumentation errors [64]. Regardless, these types of assessments were noted as vital when developing new and specific types of sensors for pressure measurements [39,68]. Several of these studies also took special care to assess whether the mechanical design and configuration of the sensors were appropriate for this unique application—in-socket measurements. For example, Lee et al., [64] and Appoldt et al., [66] presented the importance of the sensor contact area. Sensor calibration and sensor dimensions were also highly emphasized among the reviewed papers. However, only a few studies reported the known measurable pressure ranges of the sensors they used in their tests. As an example, El-Sayed et al., [68], mentioned a static force measurement range of 0–100 N for the studied system as this made it superior to several other systems in this regard.

The codes relating to this pattern are summarized in Table 7.

#### 3.2.2. Sensor Placement

Most of the studies discussed the placement of sensors in detail, often from the perspective of ensuring a systematic, repeatable placement with respect to the anatomy of the residual limb. This was related to two major discussion topics: firstly, that the value of pressure measurements depends on the location in the socket where they are recorded. For example, pressure measurements at the brim were noted to be of interest in six papers, reinforced by the fact that maximum recorded pressures were often found at the socket brim. An additional reason for the prominence was likely due to the primary use of two socket design types: brimless sockets and containment sockets. More importantly, the emphasis on the specific regions was at times driven by certain troublesome areas, which are indicative of clinically relevant sensitive regions on the residual limb [71]. Secondly, it was shown that there are differences in pressure between the proximal and distal, anterior and posterior, and medial and lateral areas of the residual limb throughout the gait cycle. One study even noted that there is a difference in the forces in the anterior–posterior direction when walking on level ground compared to an inclined surface [74].

To give an example of a systematic and repeatable method of sensor placement, Lee et al., [64] identified a plane 50 mm proximal from the distal end of the socket and another 25 mm distal from where the subject’s perineum would be positioned in the socket. Sensors were then positioned at the level of these planes and halfway between them; on the anterior, posterior, medial, and lateral mid-lines of the socket wall. A standardized naming convention of the sensors was also defined in this study.

The method of attaching sensors to the socket is considered as a factor that must be chosen carefully, as it can interfere with the original fit of the socket. However, this was only highlighted by a few papers; only two papers informed the reader that sensor integration was not a trivial task, especially to avoid co-interventions [66,69]. In addition, a single paper specifically did not allow the subject to remove the socket during the test to minimize the unintended re-positioning of sensors in the socket.

The codes relating to this pattern are summarized in Table 8.

#### 3.2.3. Data Handling and Presentation

Many papers focused on repeated tests and average or mean pressure measurements. Some of these papers also reported standard deviations, while even fewer referred to variance of the measurements. It seems natural to present average or mean measurements considering that most papers studied the ambulation of their test subjects, which repeats in cycles. A few of the papers explicitly identified repeating pressure patterns during gait. Some studies compared or justified their results with existing literature or prior knowledge. As an example, some authors referred to the expected two-peak pressure profile during walking, which is also reflected in ground reaction force profiles [39,74].

To elaborate, a few papers presented normalized pressure curves against the gait cycle, while others used phases of gaits to generalize the measured outcomes. An interesting observation was that this approach means that exact measurements are not always necessary, as much can be understood by considering the relative differences [65]. It was suggested that this could decrease the problems associated with drift and delays; for instance, [65]. Despite this focus, fewer than half of the reviewed papers noted or discussed variation to a substantial degree. Only a few studies made use of exact values in the form of the identified maximum pressure values—both with respect to the anatomical location and/or position in the gait cycle. Only two papers mentioned that pressure during static stances is also valuable. This might suggest a simplistic approach to studying relative differences.

Further complicating this approach is the temporal aspect. On the one hand, Appoldt et al., [66] performed repeated tests across multiple days, weeks, and months to ensure validity, as while the actual measurement values could differ between tests, the changes in measurements across the individual test could be shown to remain. On the other hand, Appoldt et al., [66,67] also noted that such temporal changes on participants’ residual limbs over weeks and months could mean that tests (e.g., for specific sensor locations) running over more than a few days would eventually yield unusable results.

Only Neumann et al., [65] and Appoldt et al., [67] standardized subjective measurements using a set scale. However, as the pressure and load transfer readings are not necessarily homogeneous across patient cohort groups (i.e., subject specific results), discussing subjective results can therefore be valuable. Furthermore, in some studies, subjective and objective evaluation parameters are used together.

The codes relating to this patterns are summarized in Table 9.

## 4. Synthesis and Discussion

The aim of this review was to identify the current practices of sensors in transfemoral sockets with the ultimate goal of advancing and improving prosthetic socket design, comfort, and fit for transfemoral amputees. To achieve this, two research questions came to light:

1. Is there a de facto methodology for experimental studies of the implications of intra-socket pressure sensors used by transfemoral amputees?
2. How should sensors be designed for application in prosthetic sockets to monitor transfemoral amputees towards evaluating comfort and fit?

In this section, the first question is addressed by discussing the descriptive codes in a more direct manner. Meanwhile, a holistic approach is taken with regards to the second question as it involves several different knowledge domains. Finally, the outcomes from these two research questions are concluded with considerations for future research.

### 4.1. De Facto Methodology

From the coding analysis of the literature, a few common methods were found that indicated a de facto methodology.

The importance of sensor selection was inferred by the numerous codes found relating to sensor characteristics for this application—lower-limb prosthetic sockets. A significantly large range of recorded maximum socket–residual limb interface pressures were cited across the literature. This indicates the importance of sensor calibration prior to experimental procedures, which is supported by the fact that seven of the reviewed papers reported on the calibration methods and measurable range of the sensors [39,64,65,66,67,68,71]. Care should therefore be taken to select sensors with suitable capabilities and to appropriately calibrate the sensors to enable accurate recordings over the aforementioned large pressure ranges. For comparative pressure measurements, it may be necessary for the sensors to also be sensitive to low pressures. Sensor sensitivity and a suitable error measurement range should therefore also be considered. This is particularly important if the same sensors are used for different subjects and socket types. Individual patient characteristics can significantly influence the pressure readings. For example, patient weight in combination with the size of the sensing area are obvious factors that will influence the magnitude of pressure [65]. It is therefore not only important to document the patient characteristics but also to consider their effect on the resulting intra-socket pressure distributions.

Current technological challenges and practical issues—such as sensor thickness, sensor accuracy for measurement in curved surfaces, and tethered measuring systems, as well as the highly personalized nature of prosthetic sockets—makes it unfeasible to measure dynamic pressures across the entire stump–socket interface simultaneously [77]. Although it is possible to record section-wise pressure measurements until the whole residual limb has been mapped, it is time-consuming, impractical, and leads to other variabilities that could influence the pressure mapping results, and hence also socket comfort or fitting interpretations [65]. The reviewed literature has presented various alternative solutions to address these limitations, such as methodically placing individual sensors at specified sites in the prosthetic socket. This systematic sensor placement must be based on each individual’s anatomy to ensure pressure comparisons are made in the same anatomical region for different patients. This is important as it is evident that the maximum recorded pressure values vary greatly depending on the anatomical region [64]. This approach was proven beneficial for pressure distribution comparisons across different socket types and subjects in a number of studies [64,66,67,71]. There are strong implications from the synthesized evidence of our work that more valuable pressure information is obtained when sensors are positioned at specified known problematic regions; for example, the medial brim or the distal end [64,66,67,71].

To fulfill the need for repeatable sensor placement, a number of pre-defined sensor locations could be assigned. This assignment could be standardized based on anatomical segmentation; for example, by dividing the residuum into quadrants—anterior, posterior, medial, and lateral—and further discretizing them into proximal and distal regions. Additional sensors could then be given the flexibility of being positioned at subject-specific problematic sites, such as at the brim, or in sensitive areas prone to tissue breakdown. This standardized approach enables both intra-subject and inter-subject relative pressure comparisons, reducing the importance of absolute pressure values. To elaborate, measurements at low-pressure regions could provide insights regarding the implications of comparatively high-pressure regions for the individual patient—with regards to comfort or risk of tissue damage, for example. Meanwhile, the relative pressure difference in these separate regions can be compared between subjects to eliminate the need to account for subjects’ weights.

The method of sensor integration in the socket should also be considered to avoid co-intervention. It should be ensured that the subject feels the socket fits as it would without the sensing system in place. This highlights the importance of giving the subject time to adjust to the socket prior to tests, and noting if the socket fit is unusual. An unusual fit could cause the subject to walk with an atypical gait, which could result in abnormal pressure measurements and distributions.

One reason it is difficult to compare across different studies is the almost unlimited possibilities for presenting data. The time-dependent (i.e., days, weeks, months) variability of pressure in the socket strongly suggests average measurements to be an optimal method for presenting the data. As the pressure magnitudes vary greatly depending on the anatomical region, presenting the pressure information separately for each anatomical compartment is also recommended. Segmenting and averaging measurements with respect to the gait cycle to identify cyclic pressure patterns—and reporting the standard deviations—is at least arguably more informative than simply citing the maximum measured pressure values.

**Figure 2:**
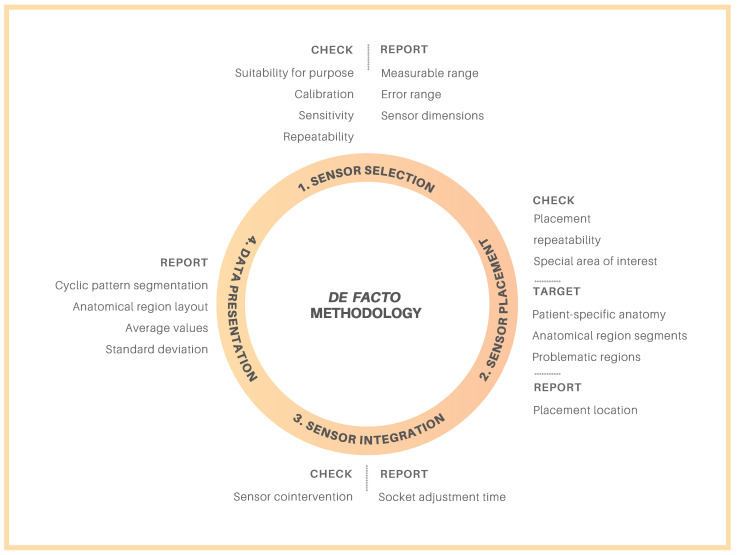
Suggested de facto methodology.

Overall, with regards to sensor implementation, a de facto methodology that was evaluated from the literature can be summarized into four major categories: (1) sensor selection, (2) sensor placement, (3) sensor integration, and (4) data presentation, as illustrated in Figure 2. Although this methodology was primarily initiated considering transfemoral prosthetic socket users, and while these categories individually play a crucial role, many attributes from each category can be directly transferred for use by patients with transtibial amputations. However, some items must be specialized depending on whether the patient has a transtibial or transfemoral amputation. Firstly, the suitability of sensors is based on the ability to integrate the hardware into the socket. This includes the method of hardware integration with the socket as well as sensor dimensions to ensure the sensors neither interfere with the rest of the prosthetic system nor cause contraindications for the patient with regards to socket comfort and fit. Secondly, ensuring the technical suitability of the sensors is also recommended. For example, the measurable pressure range should cover the expected pressure ranges in the socket during activities of daily living. Furthermore, the establishment of a sensor calibration procedure for repeatable measurements should allow for comparable measurements between patients. It is likely that sensor calibration procedures and sensitivity and repeatability requirements will be the same for measurements in both transtibial and transfemoral sockets. Conversely, the sensor dimensions required may differ depending on amputation level as transtibial sockets tend to be much smaller with smaller radii of curvature, for instance.

A method for the repeatable placement of sensors is particularly advantageous for inter-subject comparisons, but is also helpful for testing different sockets for the same subject [78]. This procedure is likely to be directly transferable between transtibial and transfemoral socket types. However, different socket types are designed to influence gait stability and interactive motions within the soft tissue, between the soft tissue and the socket [15,18,20], and femoral movement within the residual limb [79]. Furthermore, the specified anatomical locations of interest will differ between amputation levels. In transtibial sockets, for example, the tibial crest and tibial tubercle often require pressure relief [20], and thus it would be meaningful to measure pressure at these locations. However, in transfemoral sockets, sensors located at the ischium would be valuable as this can be the most problematic region for transfemoral amputees [18]. Individual patient residual limb characteristics, such as stump shape, tissue stiffness, the underlying muscle strength, and scar tissue, also significantly influence interfacial pressure patterns [67,69,73]. This further highlights the need to target sensor placement not only based on the amputation level but ideally also customized for each patient to yield valuable results.

Strategic sensor selection (as previously described) should facilitate smooth hardware integration with the prosthetic socket. However, cointerventions can be minimized further by providing the patient with an adjustment period and by requesting feedback should their socket feel unusual.

Presenting relevant data in a coherent manner is essential in order to provide valuable information to the scientific community. The literature revealed the advantages of segmenting pressure into gait cycles and citing the average values. Given the contrasting pressure ranges and patterns depending on the anatomical location, the benefits of displaying the findings with respect to the patient’s anatomy was also highlighted from the reviewed literature. Furthermore, it is highly recommended that future studies record subjective feedback from the patients themselves.

The suggested de facto methodology is based on the studied literature, but as should be apparent, no single study makes use of all of its parts. This paper can thus highlight attributes that can be transferred from and for use by patients with transtibial amputations and others which require specialization for patients with transfemoral amputations. However, we must conclude that directly comparing pressure measurements between the included studies, or to measurements taken in studies including patients with transtibial amputations, cannot (yet) be justified. The characteristics of patients are seldom described well enough to ensure valid comparisons, and when these data are available, there is substantial variation in regard to important characteristics such as weight. In fact, none of the included studies broached the subject of known gait deviations due to the improper construction of transtibial and transfemoral prostheses. The impact of these on pressure readings can vary substantially, as they can lead to an exaggerated transfer of load to a patient’s healthy side and involve effects such as foot slaps [80]. Sensor characteristics such as measurement and error range would most likely be most important when identifying such gait deviations with precision. However, the total error range of pressure sensors is, for example, seldom discussed, and when discussed, this is only in comparison to other sensor technologies. This does not help to define how large the total sensor error can be and remain acceptable, neither in the generic case nor for any specific patient type or gait deviation.

Hopefully, with more studies adhering to all parts of the suggested methodology, the acceptable and suitable ranges for measurements and errors can be identified.

### 4.2. Implications for the Design of Sensors

The implications of this survey for how sensors should be designed for application in prosthetic sockets of transfemoral amputees for monitoring to evaluate comfort and fit is strongly tied to what the discourse does not contain; almost all surveyed papers avoided defining hypotheses in lieu of large-scale data collection for inductive analysis.

Inductive analysis can be a powerful tool, and it would be speculative to attribute its widespread use among the papers to any particular motivation of the authors. Rowbottom and Alexander [81] found that exploratory data gathering is actually more widespread in biomechanical research than a quick reading would imply. In other words, presentational hypotheses—i.e., hypotheses that do not influence the actual study—seem to be a frequent problem in the field. As this practice can be misleading regarding the significance of results, there is most likely a need for researchers within the field to start accepting such studies on their own merits (to avoid tempting researchers to present their studies as something they are not). However, exploratory data gathering seems to be especially difficult to use for designing sensors for monitoring to evaluate comfort and fit. Feelings of comfort or discomfort attributed to a well-fitting or ill-formed prosthesis can be influenced by many characteristics of the amputee, prosthesis, and surrounding environment. Generalizing across large amounts of data is easily confounded by such factors, as they for instance mean that comfort is not directly correlated to tightness or looseness [73] and that discomfort can be “disguised” by an abnormal gait [72]. Similarly, this means that the data are not necessarily suitable for analysis, as these characteristics can skew data sets. For example, both highly contoured sites on the residual limb [34] and the way the environment in a prosthetic exposes sensors to wear [34,69] can confound measurements. To properly validate a sensor system, several different types of scenarios might have to be considered—a potentially costly requirement. As an example, a recently developed sensor system was validated in three scenarios: (1) a flat bench-top test, (2) in a prosthetic socket at a low curvature site, and (3) in a socket at a higher curvature site [78]. In fact, the same sensors were also tested under dynamic cyclic loading to simulate realistic use conditions.

It is understandable if the sheer complexity provokes researchers working on sensor technology to focus more on technology and less on contextual factors (see e.g., [69]). However, this limited perspective is likely an obstacle to innovation in sensor design in this context. Contemporary studies by researchers that actually use pressure sensors to investigate monitoring to evaluate comfort and fit have more specific needs. These include, for instance, attempts to identify whether amputees tense their muscles to avoid high pressure [64]; the size of areas of local, high pressure [65]; prosthetic migration and socket movement [71]; and whether the prosthesis stabilizes the residual limb [73]. Large-scale data measurements of pressure at several locations *could* by pure chance help to solve these needs, but it is highly unlikely. To help address this issue, we propose guidance in three steps for researchers targeting this application domain.

1. Define and operationalize hypothetical constructs: Fit and comfort are hypothetical constructs—i.e., qualitative variables—which are not directly observable. Unfortunately, with no agreed way of operationalizing these constructs for transfemoral amputees, the implications of any pressure measurements are unclear. Therefore, sensor designers should ensure that their pressure measurements can be related to a suitably limited part of fit and comfort—ideally, with the support of experts possessing relevant knowledge on the constructs.
2. Ensure necessary coverage of the operationalized construct: This suggests that it would be fruitful to develop pressure sensors in close combination with other types of sensors. Otherwise, it might be difficult to ensure that the operationalization can be properly measured. As an example, several of the surveyed papers noted an interest in measuring shear [39,72,74]. Presumably, a well-integrated combination of pressure and shear sensors is required to answer several of the aforementioned needs, such as identifying the existence, explanation, and effect of prosthetic migration on fit. The integration of other types of sensors with pressure sensors can provide a deeper understanding of the interaction between the residual limb and the prosthetic socket. For example, electromyography (EMG) [82] and temperature sensors [83] can provide insights to the relationship between muscle activities and intra-socket climate, respectively, with interface pressure. The integration of gait monitoring devices is undoubtedly useful to evaluate these sensor measurements as a function of the gait cycle, or with respect to level of activity [4,9,32,78,82]. This might seem trivial to accomplish, but there are several reasons why researchers are currently avoiding this approach. To name a few, it requires knowledge of several types of sensor technology, it multiplies the difficulty of all problems related to handling large amounts of data, and it makes issues related to advanced prototyping even more likely to surface.
3. Make use of relevant expertise when evaluating measurements: Finally, as achieving correct operationalization and measurement coverage are difficult, results must (also) be evaluated together with experts possessing relevant knowledge on the constructs. This is especially important as results might otherwise only be considered in relation to the most obvious use cases; i.e., level ground walking. As an example, not much effort has been devoted to studying the implications of sensor measurements for the wearing of prostheses, even though the pressures measured during this use case can be as high as during normal walking [67]. Naturally, professionals working with prostheses can be uninterested in adopting pressure sensors, or risk misunderstanding their implications, if pressure readings are only fully understood for a small part of normal prosthesis usage.

If researchers who wish to have an impact on the medical application domain adopt these three steps during conceptual design and pressure sensor evaluation, the chance of breakthroughs in prosthetic design should increase substantially.

### 4.3. Implications for Research

It is clear that experts from several different domains need to cooperate in a consistent way to carry out the identified de facto methodology, especially when the aim is to achieve breakthroughs in sensor design. Two examples of when cooperation is required are as follows: (a) even if engineers are the foremost experts on identifying appropriate sensor capabilities and calibration, this activity will rely on prosthetists’ knowledge regarding prostheses and medical experts’ knowledge of patients; and (b) even if medical experts can identify problematic regions on stumps, recording pressure there requires engineers to develop portable, and yet powerful, data recording systems. As an example, Karamousadakis et al., [84] developed a fuzzy logic-based decision support system for socket design modifications for improved fit. The fuzzy rules are based on the expert knowledge of prosthetist(s), and the patient-specific socket modification suggestions are governed by their own intra-socket pressure information. Furthermore, as noted, overcoming complexity and focusing on the most important questions will require all of these experts to share their domain knowledge with each other.

This study has two important implications in this regard. Firstly, the de facto methodology can form a base for comparing studies. However, it can also be help to make studies valuable to a wider group of experts. Dividing pressure data between anatomical regions might not be important for the purpose of an engineer designing a novel sensor, but it might be critical for a prosthetist to make further use of the study. Secondly, purely inductive, data-driven research might not be optimal for achieving breakthroughs in this area. Ideally, this should be solved by more hypothesis-driven research in each study through close cooperation between different domain experts. However, when this is not possible, researchers should consider creating fine-grained open research data to possibly support the hypotheses testing of other, future studies.

## 5. Conclusions

This paper has presented the results from a scoping review of the existing research on pressure measurements in prosthetic sockets worn by transfemoral amputees during ambulation.

The high-quality literature focusing on this topic has been produced over several decades and by many different research groups. Nevertheless, a de facto, best-practice methodology for experimental studies on this topic can be identified.

The survey identified that the most in-depth results were found by investigations that chose to define hypotheses rather than conduct large-scale data collection for inductive analysis. Many factors come together that make it unlikely that important discoveries will be found contingently though traditional data-processing. This suggests that sensors for application in prosthetic sockets of transfemoral amputees to monitor and evaluate comfort and fit need to be designed to fulfill well-defined goals. To support research on this topic, sensor developers should thus consider designing combinations of sensors and seek out clinical experts in the field to enable valid research on both common and critical, rare-use cases.

Furthermore, both the de facto methodology and ways to improve sensor design identified by this study suggest that experts from different domains should work in closer collaboration. To further excel in the field, researchers should try to report fine-grained open research data in relation to the de facto methodology, possibly even to support other studies by considering corner cases that are not of primary interest to themselves.

## Figures and Tables

**Table 1:** Keywords searched.

#	Keywords	Databases Searched
1	transfemoral AND socket AND pressure	Science Direct, Web of Science, PubMed, Scopus
2	socket AND pressure	PubMed, Scopus
3	transfemoral AND stump AND pressure	Science Direct, Web of Science, PubMed, Scopus
4	“above knee” AND socket AND pressure	Science Direct, Web of Science, PubMed, Scopus
5	“above knee” AND stump AND pressure	Science Direct, Web of Science, PubMed, Scopus
6	prosthetic AND socket AND pressure	PubMed, Scopus
7	“prosthetic socket” AND pressure	Science Direct, Web of Science, PubMed, Scopus
8	transfemoral AND “residual limb” AND pressure	Science Direct, Web of Science, PubMed, Scopus
9	“above knee” AND residuum AND pressure	Science Direct, Web of Science, PubMed, Scopus

**Table 2:** Quality Assessment Table (QAT).

Category	Title	Description
Hypothesis	Definition of investigation	Identifies if the hypothesis/aim/objective of the study is clearly defined
Patient Selection	Adequacy of description of inclusion and exclusion criteria	Tests sufficiency of patient sample definition *
	Functional homogeneity	Tests homogeneity of patient sample **
Intervention and Assessment	Experimental intervention	Tests sufficient information on experimental intervention description ^†^
	Cointerventions	Tests whether cointerventions were avoided or were compared and accounted for ^††^
	Timing of measurements	Identifies whether patients were given enough time to adapt to the prosthesis
	Outcome measures	Identifies if the outcome parameters have been clearly defined ^‡^
Statistical Validity	Dropouts	Identifies sufficient reporting on number of dropouts and reasons for dropouts
	Intention to treat	Identifies sufficient assessment on intention to treat analysis in the case of dropouts
	Data presentation	Tests presentation of primary outcome measures ^‡‡^

* Participant characteristics are sufficiently detailed for future replication; minimum of three out of patient evaluation, residual limb evaluation, and anthropometric measurement criteria (e.g., age, weight, gender, level of amputation, socket type, activity level, time since amputation, residual limb condition) ** Homogeneous patient sample (e.g., at least the level of amputation and activity level of included subjects should be reasonably equal) ^†^ Sufficient explanation of a minimum of three experimental intervention criteria (e.g., sensor type, number of sensors, sensor placement, socket type, gait kinematics and kinetics, walking conditions) ^††^ For single-subject studies, experimental intervention must be clearly defined and steps taken to ensure the experiment represents their normal prosthesis/gait as closely as possible, or well-explained otherwise. For multi-subject studies, the results of differing experimental interventions must be discussed; e.g., if subjects are wearing different socket types, the effects of this must be discussed. This may include patient characteristics, such as gait type, age, stump condition, etc. ^‡^ Identifies whether the outcome measures have been collected using a standardized protocol (at least one from pressure vs. gait cycle or pressure vs. physical distribution) ^‡‡^ Statistical validity for adequate point estimation and measures of variability (e.g., statistical analysis and/or explicit numerical data range stated).

**Table 3:** Grading system.

Grade	Criteria
A	Minimum 8 points in total AND fulfills category X, category A, B5, and B9
B	Minimum 7 points in total AND fulfills category X, A1, and B9, OR minimum 9 points in total
C	Minimum 5 points in total
D	Less than 5 points in total

**Table 4:** Papers selected for data synthesis and their grades.

Author, Year	Publication Title	Grade
Lee et al., 1997 [64]	Stump–socket interface pressure as an aid to socket design in prostheses for trans-femoral amputees—A preliminary study	A
Neumann et al., 2005 [65]	Concepts of Pressure in an Ischial Containment Socket: Measurement	A
Appoldt et al., 1967 [66]	A preliminary report on dynamic socket pressures	B
Appoldt et al., 1968 [67]	Stump–socket pressure in lower extremity prostheses	B
El-Sayed et al., 2014 [68]	Piezoelectric bimorphs’ characteristics as in-socket sensors for transfemoral amputees	B
Ferreira et al., 2017 [69]	Piezoresistive Polymer-Based Materials for Real-Time Assessment of the Stump/Socket Interface Pressure in Lower Limb Amputees	B
Hong et al., 2006 [70]	Effect of hip moment on socket interface pressure during stance phase gait of transfemoral amputee	B
Kahle et al., 2013 [71]	Transfemoral interfaces with vacuum assisted suspension comparison of gait, balance, and subjective analysis: Ischial containment versus brimless	B
Laszczak et al., 2016 [39]	A pressure and shear sensor system for stress measurement at lower limb residuum/socket interface.	B
Leavitt et al., 1972 [72]	Gait analysis and tissue–socket interface pressures in above-knee amputees	B
Naeff et al., 1980 [73]	Dynamic pressure measurements at the interface between residual limb and socket—the relationship between pressure distribution, comfort, and brim shape	B
Tang et al., 2017 [74]	A combined kinematic and kinetic analysis at the residuum/socket interface of a knee-disarticulation amputee	B

**Table 5:** The importance of biomechanical hypotheses.

Code	Papers
The importance of biomechanical hypotheses	[64,65,71,73]

**Table 6:** References to domain knowledge.

Code	Papers
Wearing stress	[67]
High pressure does not equal discomfort	[73]
Challenges for sensors	[69]
Shear of interest	[39,72,74]
Test subject profile given	[39,64,67,68,70]
Walking speed defined	[39,64,65,68,70,71]
Prosthesis defined	[39,64,65,67,70]
Accustomed to socket type	[64,65,68,71]
Residual limb assessed	[65,70]
Accustomed to socket under test	[66,67]
Test subject warm-up	[64,65,70,71]
Avoid fatigue	[64,66,67]

**Table 7:** Assessment of sensor type and use.

Code	Papers
Assessment of sensor application	[39,64,65,66]
Assessment of sensor type	[39,64,65,66,67,68,69]
Known error range of sensors	[39,64,65,66,67,68]
Known measurable range of sensors	[39,66,68]
Sensors calibrated	[39,64,65,66,67,68,71]
Sensor dimensions mentioned	[39,64,65,68,71]

**Table 8:** Sensor placement.

Code	Papers
Systematic and repeatable sensor placement	[64,65,66,67,69,73,74]
Systematic naming convention of sensors	[64,67]
Sensor placement locations noted	[39,64,65,67,68,71,72]
Brim especially important area	[39,64,66,67,71,72,73]
Special areas of interest	[64,65,66,67,71,72,73]
Proximal vs distal	[39,64,65,66,67,68,72,73]
Anterior vs posterior	[39,66,67,68,69,70,74]
Medial vs lateral	[66,69,72]
Method of sensor attachment described	[64,66,67]
Non-trivial integration of sensors	[66,69]
Avoid socket doffing	[64]

**Table 9:** Data handling.

Code	Papers
Repeated tests	[64,66,67,68,70,74]
Measurements averaged	[39,64,65,66,70,71,72,73,74]
Standard deviation mentioned	[39,64,65,66,67,71]
Variation mentioned	[39,64,66,71,74]
Repeated pressure patterns identified	[39,64,65]
Validation against previous studies	[64,65,68,71]
Two peak pressure profiles identified	[39,64,68,73,74]
Pressure compared with gait phases	[66,67,70]
Relative measurements emphasized	[65,66]
Maximum pressure mentioned (Cf. gait cycle)	[64,65,66,67,70,71,73]
Maximum pressure mentioned (Cf. anatomical location)	[39,64,65,71,73]
Static stance pressure important	[66,71]
Tested at time intervals (days/weeks/months)	[66]
Comparison over long time undesirable (days/weeks/months)	[66,67]
Subjective measurements standardized	[65,67]
Subject-specific results presented	[64,67]
Subjective and objective results discussed	[64,65,67,71,72,73]

## Data Availability

Not applicable.

## Abbreviations

The following abbreviations are used in this manuscript:

PEQ	Patient evaluation questionnaire
PRISMA	Preferred Reporting Items for Systematic and Meta-Analyses
FEA	Finite element analysis
QAT	Quality assessment table

## Appendix A. Papers Excluded after Quality Assessment

**Table A1:** Papers excluded after QAT.

Author, Year	Main Reason for Exclusion	Unfulfilled Criteria	Grade
Appoldt et al., 1970 [85]	Not well-defined definition of the investigation	X0, A2, B6, C13	C
Hong et al., 2005 [82]	Insufficient description of study population	A1, A2	C
Tanaka et al., 1997 [86]	Insufficient description of study population	X0, A1, A2, B6, B8, C13	C/D
Colombo et al., 2016 [87]	Insufficient description of study population	X0, A1, A2, B6, B8, C10, C12, C13	D
Moineau et al., 2015 [88]	Insufficient description of study population	A1, B8	C/D
